# 504. Individual and Neighborhood Factors Associated with Failure to Vaccinate Against COVID-19 Among Pregnant Persons

**DOI:** 10.1093/ofid/ofad500.573

**Published:** 2023-11-27

**Authors:** Ousseny Zerbo, Thomas Ray, Bruce Fireman, Evan Layefsky, Kristin Goddard, Pat Ross, Mara Greenberg, Nicola P Klein

**Affiliations:** Division of Research Kaiser Permanente Vaccine Study Center, Oakland, California; Kaiser Permanente Northern California, Oakland, California; Division of Research Kaiser Permanente Vaccine Study Center, Oakland, California; Division of Research Kaiser Permanente Vaccine Study Center, Oakland, California; Division of Research Kaiser Permanente Vaccine Study Center, Oakland, California; Kaiser Permanente Vaccine Study Center, Oakland, CA; Kaiser Permanente Northern California, Oakland, California; Kaiser Permanente Northern California, Oakland, California

## Abstract

**Background:**

Pregnant persons are at increased risk of complication from COVID-19. Vaccine coverage among pregnant persons has been low. The overall goal of this study was to determine whether unvaccinated pregnant persons cluster geographically and to assess individual and neighborhood factors associated with failure to vaccinate.

**Methods:**

The study setting was Kaiser Permanente Northern California (KPNC), an integrated healthcare delivery organization that provides comprehensive care to approximately 4.4 million members. This study included a cohort of pregnant persons who delivered between December 15, 2020 and September 30, 2022. Pregnant persons were considered vaccinated if they received mRNA COVID-19 vaccines any time before or during pregnancy. We used spatial scan statistics to identify geographical clusters of unvaccinated pregnant persons and determined individual and neighborhood factors associated with failure to vaccinate using multivariate logistic regression.

**Results:**

Among 85,852 pregnant persons residing in Northern California, 46.6% were unvaccinated. Spatial analysis identified 5 clusters with high prevalence of unvaccinated pregnant persons. The proportion of unvaccinated ranged from 53% to 62% within clusters compared with 39% outside clusters. In covariate-adjusted analyses, residence in a cluster decreased the odds of vaccination by 1.64 (95% confidence interval (CI): 1.59 - 1.69). The odds of vaccination also decreased with black race (OR=1.45, 95% CI: 1.37 - 1.54), subsidized insurance (OR = 1.32, 95% CI: 1.26 – 1.38) and living in neighborhoods with high proportion of adults with high school education or less. The Odds of vaccination decreased by 1.21 (95% CI: 1.15 – 1.26), 1.36 (95% CI 1.29 – 1.44) and 1.43 (95% CI 1.34 – 153) for pregnant persons living in neighborhoods where the proportion of adults with high school education or less was 20% to < 30%, 30% to < 43% and ≥43%, respectively.

**Conclusion:**

Almost half of pregnant persons, members of Kaiser Permanente in Northern California, were unvaccinated against COVID-19. Unvaccinated pregnant persons clustered geographically and by some sociodemographic factors. Interventions to increase COVID-19 vaccine coverage among pregnant persons are needed, particularly among vulnerable pregnant populations.

**Disclosures:**

**Nicola P. Klein, MD, PhD**, GlaxoSmithKline: Grant/Research Support|Merck: Grant/Research Support|Pfizer: Grant/Research Support|Sanofi Pasteur: Grant/Research Support

